# Inter-eye genomic heterogeneity in bilateral retinoblastoma via aqueous humor liquid biopsy

**DOI:** 10.1038/s41698-021-00212-0

**Published:** 2021-07-27

**Authors:** Elyssa Y. Wong, Liya Xu, Lishuang Shen, Mary E. Kim, Ashley Polski, Rishvanth K. Prabakar, Rachana Shah, Rima Jubran, Jonathan W. Kim, Jaclyn A. Biegel, Xiaowu Gai, Peter Kuhn, James Hicks, Jesse L. Berry

**Affiliations:** 1 The Vision Center, Children’s Hospital Los Angeles, Los Angeles, CA USA; 2 USC Roski Eye Institute, Keck School of Medicine of USC, Los Angeles, CA USA; 3 Department of Biological Sciences, Dornsife College of Letters, Arts, and Sciences, University of Southern California, Los Angeles, CA USA; 4 Center for Personalized Medicine, Department of Pathology and Laboratory Medicine, Children’s Hospital Los Angeles, Los Angeles, CA USA; 5 Department of Molecular and Computational Biology, University of Southern California, Los Angeles, CA USA; 6 Cancer and Blood Disease Institute, Children’s Hospital Los Angeles, Los Angeles, CA USA; 7 Department of Pathology and Laboratory Medicine, Keck School of Medicine of USC, Los Angeles, CA USA; 8 Norris Comprehensive Cancer Center, Keck School of Medicine of USC, Los Angeles, CA USA; 9 Department of Aerospace and Mechanical Engineering, Viterbi School of Engineering, University of Southern California, Los Angeles, CA USA; 10 Department of Biomedical Engineering, Viterbi School of Engineering, University of Southern California, Los Angeles, CA USA; 11 Department of Biochemistry and Molecular Medicine, Keck School of Medicine of USC, Los Angeles, CA USA; 12 The Saban Research Institute, Children’s Hospital Los Angeles, Los Angeles, CA USA

**Keywords:** Eye cancer, Cancer genomics, Tumour heterogeneity, Translational research

## Abstract

Germline alterations in the *RB1* tumor suppressor gene predispose patients to develop retinoblastoma (RB) in both eyes. While similar treatment is given for each eye, there is often a variable therapeutic response between the eyes. Herein, we use the aqueous humor (AH) liquid biopsy to evaluate the cell-free tumor DNA (ctDNA) from each eye in a patient with bilateral RB. Despite the same predisposing germline *RB1* mutation, AH analysis identified a different somatic *RB1* mutation as well as separate and distinct chromosomal alterations in each eye. The longitudinal alterations in tumor fraction (TFx) corresponded to therapeutic responses in each eye. This case demonstrates that bilateral RB tumors develop separate genomic alterations, which may play a role in tumorigenesis and prognosis for eye salvage. Identifying these inter-eye differences without the need for enucleated tumor tissue may help direct active management of RB, with particular usefulness in bilateral cases.

## Introduction

Retinoblastoma (RB) is an eye cancer that forms in the developing retina and affects approximately 8,000 infants and toddlers each year worldwide^1^. If untreated, RB is fatal, however overall survival rates for RB in the United States have risen above 97%^2^. While mortality is improving, morbidity remains high; these children frequently sustain a loss of vision or loss of the eyes to prevent the extraocular spread of disease^3,4^. This morbidity is even more impactful in children who have a hereditary diseases with both eyes affected. This rare neoplasm occurs as a result of biallelic inactivation of the *RB1* tumor suppressor gene^5^. In hereditary RB, which accounts for approximately 45% of RB cases^1^, the first *RB1* pathogenic mutation is a constitutional germline or mosaic mutation that arises during gametogenesis or early fetal development. This germline mutation thus predisposes the patient to develop bilateral RB, as a secondary somatic mutation in any retinal cell initiates tumorigenesis. In the remaining non-hereditary cases, RB is caused by a somatic mutation, leading to biallelic *RB1* loss in the tumor only.

Landmark discoveries in the genetic origins of RB have revolutionized the field of cancer biology and, in this century, spurred an era of genomic testing and precision oncology. In 1971, Knudson elegantly described the genetic mechanism of RB with his “two-hit hypothesis”.^5^ This ultimately led to the description, localization, and cloning of the first tumor suppressor gene, which we today recognize as *RB1*. Ironically, despite the pioneering role RB has played in cancer genomics, the children who are affected by RB have not received many of the benefits that precision oncology has to offer. Unlike that of other tumors, a direct biopsy of RB is contraindicated due to the risk of extraocular tumor spread^6^. Thus, tumor tissue is only available from enucleated (surgically removed) eyes. Therefore, RB genomic biomarkers are not routinely available from eyes undergoing therapy with the goal of organ salvage.

Without access to genomic biomarkers, ocular oncologists strictly rely upon clinical features for therapeutic decisions. Despite the same predisposing germline *RB1* mutation and similar treatment, there is frequently a variable therapeutic response between each eye of the same patient. This is likely due to differences at the molecular level between the tumors in each eye. It is known that after inactivation of the second *RB1* allele, additional genomic events, including large-scale somatic copy number alterations (SCNAs), are responsible for further tumorigenesis^7^. Winter et al. recently reported distinct genomic profiles between tumors from enucleated eyes in two patients with bilateral RB^8^. Additionally, Davies et al. described enucleated bilateral tumors from one patient as having the same germline *RB1* mutation but differing somatic *RB1* mutations^9^. However, further characterizations of independent heterogenous inter-eye events in bilateral RB patients are limited given 1) the goal of avoiding bilateral enucleation and 2) the aforementioned contraindication to direct tumor biopsy of RB^6^. To overcome this problem, we utilize the aqueous humor (AH), the clear fluid that circulates in the front of the eye, as a liquid biopsy for RB. We have demonstrated that the AH is a rich source of cell-free tumor-derived DNA (ctDNA) that captures the genomic landscape of RB tumors, including *RB1* mutations and RB SCNAs^10,11^. Herein, we utilized the AH liquid biopsy to provide a comparison of genomic profiles between eyes in a patient with bilateral RB without direct tumor biopsies or upfront enucleation; we also show that ctDNA longitudinal dynamics corresponded to therapeutic response.

## Results

### Case report

**Fig. 1:**
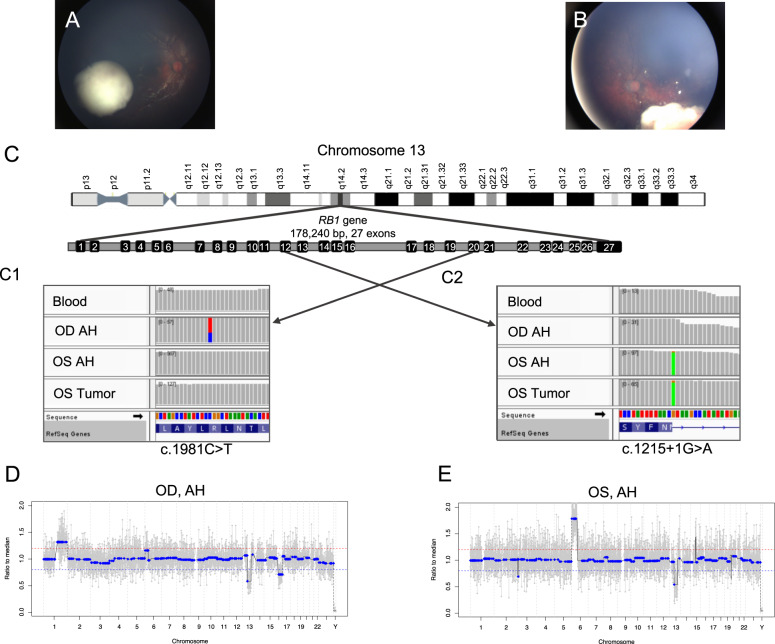
Comparison of clinical images at diagnosis and genomic profiles between right and left eyes. **A** Fundus photograph of the right eye shows a cohesive, creamy white endophytic mass with predominantly scattered dust-like seeds overlying the apex and a few spherical vitreous seeds at the base of the tumor, consistent with IIRC Group C retinoblastoma. **B** Fundus photograph of the left eye shows a creamy white endophytic mass with intratumoral vasculature, diffuse large spherical seeds, and some dust-like seeds, consistent with IIRC Group D retinoblastoma. **C** Integrative genomics viewer (IGV) displays the somatic *RB1* pathogenic variants in the right and left eyes. The *RB1* gene is located on chromosome 13 and has 178,240 base pairs with 27 exons. Here, each vertical bar represents one base pair and gray color indicates there is no change compared with the human reference genome (hg19). **C1** The right eye demonstrated a missense mutation (c.1981C>T) in exon 20, seen as the red-and-blue bar found only in the OD AH sample. This represents the second hit unique to the right eye; it is not present in the blood, OS AH, or OS tumor samples. **C2** The left eye demonstrated a splice donor variant mutation (c.1215+1G>A) in exon 12, seen as the matching green bars in the OS AH and the OS Tumor samples. This mutation is unique to the left eye and is only seen in OS samples; it is not present in the blood or OD AH samples. The right eye (**D**) and left eye (**E**) demonstrate non-identical somatic copy number alteration (SCNA) profiles. The right eye demonstrated 1q gain, 13q loss (germline), and 16q loss. The left eye demonstrated 6p gain and 13q loss (germline). Of note, the 6p peak amplitude seen in the right eye remains below the 20% deflection threshold (represented by the red line) to be considered a true gain.

This study included a total of 5 AH samples (3 samples from the right eye and 2 samples from the left eye) from a patient with bilateral RB. Patient demographics are presented in Supplementary Table 1. The right eye was classified as International Intraocular Retinoblastoma Classification (IIRC) Group C^12^/Stage cT2b^13^; the left eye was Group D/Stage cT2b (Fig. 1A, B). Focal consolidation with laser and cryotherapy was used in conjunction with primary intravenous chemotherapy per established protocol^14^. Given persistent vitreous seeding in the right eye, three intravitreal melphalan (IVM) injections were performed. All seeds resolved, and the right eye was salvaged without subsequent recurrence over a total follow-up of 32 months. The left eye also had persistent seeding after primary chemotherapy and was treated with two IVM injections and one intravitreal topotecan injection. Despite this therapy, the left eye developed multiple areas of tumor recurrence in the retina and vitreous cavity, requiring subsequent enucleation to prevent extraocular tumor spread 10 months after diagnosis.

### *RB1* mutation detection

In peripheral blood, a large-scale 39Mbp 13q deletion (including the *RB1* locus at 13q14.2) and focal 949kbp loss of 16p12.2 were detected. *RB1* mutational analysis of AH ctDNA from the right eye identified the 13q deletion and the second somatic mutation: c.1981C>T (p.R661W), a missense mutation, with a variant allele frequency (VAF) of 31% (Fig. 1C1). Analysis of AH ctDNA from the left eye demonstrated a different somatic mutation: c.1215+1G>A, a splice donor variant, with a VAF of 92.6% (Fig. 1C2). This somatic *RB1* mutation was similarly identified in the matched tumor sample obtained after enucleation of the left eye (tumor VAF 92%).

### Whole genomic analysis of AH ctDNA

Three SCNAs (1q gain, 13q loss, and 16q loss) were identified in the right eye and two SCNAs (6p gain and 13q loss) were detected in the left eye (Fig. 1D, E). The only shared alteration between eyes was the known germline 13q loss (Supplementary Fig. 1). Of note, the germline focal 16p deletion seen on peripheral blood testing was not detected in SCNA profiling of either eye, as it was below our 1Mbp detection threshold with ultra-shallow whole genome sequencing and copy number profiling. No pathogenic *BCOR* or *CREBBP* mutations were detected in this patient.

### Tumor fraction

**Fig. 2:**
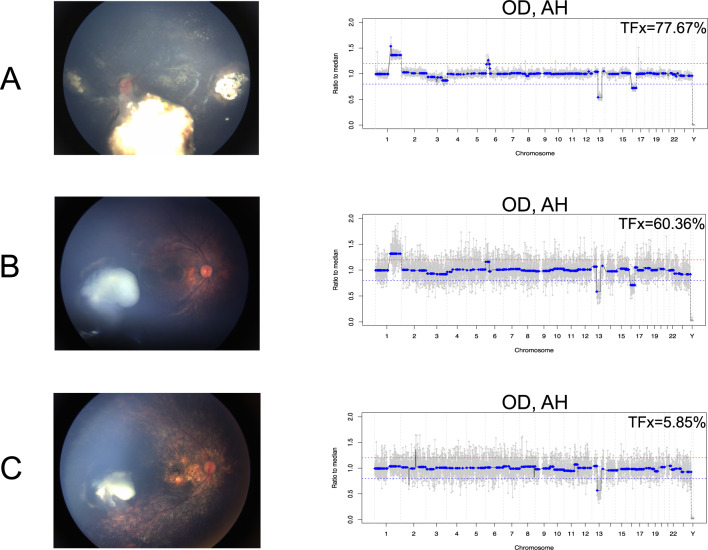
Fundus photos of the right eye and matching genomic profiles from the aqueous humor (AH) obtained at the time of intravitreal melphalan (IVM) injections. In the right eye, tumor fraction (Tfx) steadily decreased over the course of 3 IVM injections (**A**, **B**, and **C**) and the TFx percentage calculated by ichorCNA is shown in the top of each genomic profile. The decreases in TFx are accompanied by decreased amplitude of SCNAs, as expected, and decreased tumor burden shown in the matched fundus photos. Of note, the 13q deletion is a germline chromosomal alteration and its amplitude is not affected by decreased TFx.

**Fig. 3:**
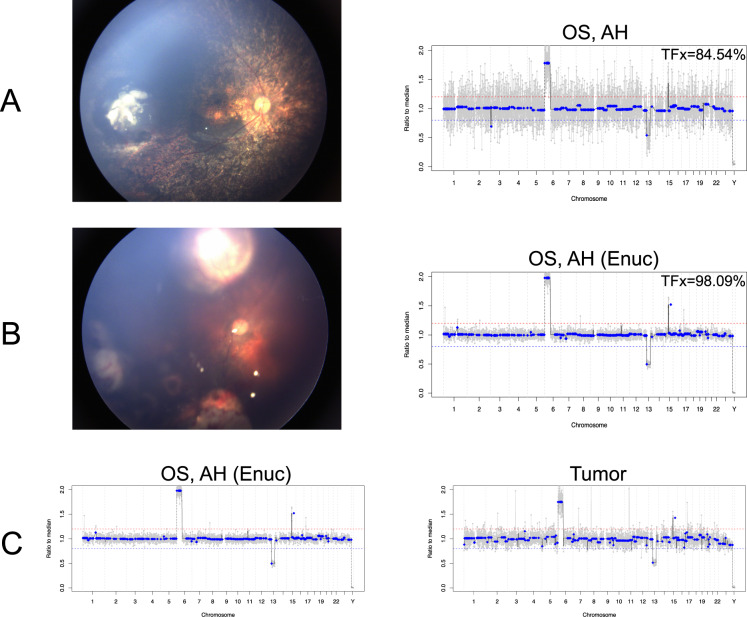
Fundus photos of the left eye are shown alongside matched genomic profiles. **A** The left eye demonstrated a high tumor fraction (TFx) of 84.54% during intravitreal chemotherapy corresponding to the active vitreous seeds seen clinically. **B** Enucleation was performed due to an active retinal recurrence shown in the fundus photo. Immediately after enucleation of the left eye, TFx from AH was 98.09%. **C** AH obtained immediately the following enucleation and matched tumor tissue demonstrated 99.79% concordance in the presence of genomic alterations.

In the right eye, tumor fraction (TFx) in the AH decreased from 77.67% to 5.85% over the course of 3 IVM injections (Fig. 2). The left eye demonstrated an increase of AH TFx from 84.54% during intravitreal treatment to 98.09% at the time of enucleation (Fig. 3A and B).

### AH and tissue concordance

Consistent with previous results^10,15,16^, genomic profiles from 1) AH obtained immediately following enucleation and 2) matched tumor tissue in the left eye demonstrated 99.79% concordance (Fig. 3C).

## Discussion

Herein, we demonstrate distinct inter-eye genomic profiles in a case of bilateral RB with the same predisposing germline 13q deletion. This heterogeneity clearly illustrates that RB tumorigenesis progresses independently in each eye, adding to recently published studies of patients with bilaterally enucleated RB^8,9^. Using an AH-based liquid biopsy avoids the need for enucleated tumor tissue and enables longitudinal monitoring of molecular tumor dynamics during active globe-conserving therapy for each eye. Implementation of the AH liquid biopsy offers great potential for precision management of this pediatric cancer, specifically with regards to tailoring therapeutic strategies for each eye in patients with bilateral RB.

Larger AH liquid biopsy studies have shown that chromosome 6p gain–specifically with greater than 1.5 median amplitudes of gain-is significantly associated with increased risk of enucleation^15,16^. In this case, the left eye had a pronounced 6p gain with an amplitude of 1.8 and ultimately required enucleation due to failed response to therapy; however, the right eye, which did not have a 6p gain, was successfully treated and saved (Fig. 1D). For patients with bilateral RB, a prognostic biomarker like 6p gain is only useful if each eye can be investigated separately. Hence, an advantage of the AH liquid biopsy over any blood-based approach is its ability to evaluate ctDNA in each eye individually and provide eye-specific prognostication in the setting of bilateral disease^15,16^.

Blood-based liquid biopsies for RB have been discussed, yet successful identification of RB-derived ctDNA in blood has only been described in instances of locally advanced or metastatic disease and at tumor fractions too low for the identification of SCNAs^17^. In contrast, we have shown that both pathogenic *RB1* mutations and SCNAs are detectable in the ctDNA isolated from 100 μL of AH and can be analyzed in each eye separately^11^.

As a further application of the AH liquid biopsy to precision medicine for RB, these findings suggest that an AH liquid biopsy can be used to longitudinally trend TFx, which corresponds to treatment response. Based on studies of non-ocular cancers^18,19^, as well as AH in RB^20^, a decrease in TFx corresponds to disease regression, as seen in the right eye of this case. There was a dramatic decrease in TFx from 77.67% to 5.85% seen in the right eye that was observed with decreased vitreous seeding and shrinkage of the main tumor with a positive therapeutic response. This decrease in TFx correlated with a decrease in the absolute SCNA amplitude of the somatic alterations 1q and 16q, but the amplitude of 13q was not affected as the latter is a germline alteration that is not solely in the tumor and thus the amplitude of loss is not affected by TFx. This constellation of findings is consistent with our previous publication that demonstrated longitudinal changes in AH-derived cfDNA TFx and SCNA amplitude corresponding to clinical responses during active therapy^20^. In contrast, the left eye demonstrated an increase in TFx associated with tumor recurrence. Because TFx changes correlate with disease activity^18–21^, implementing an AH liquid biopsy platform could offer an objective way to monitor therapeutic response in each eye independently and detect recurrence before they are clinically visible in RB eyes.

In this study, AH obtained immediately following enucleation and matched tumor tissue in the left eye demonstrated 99.79% concordance. However, in the setting of multifocal disease due to germline *RB1* mutation, each tumor clone may develop different SCNAs, yielding a blended AH ctDNA profile that may differ from the matched tumor profile^10^. Further research is necessary to determine if there is intra-eye genomic heterogeneity in cases of multifocal disease, and if so, how the AH liquid biopsy may be best applied in these cases.

Herein, we present a report of separate, distinct genomic profiles derived from AH ctDNA in each eye of a patient with bilateral RB. An AH liquid biopsy platform that can be implemented during active treatment has promising advantages for RB management, with particular usefulness in bilateral disease. Our strategy of using the AH as a liquid biopsy may provide the groundwork for further investigation and identification of additional biomarkers which could lead to the development of more precise intervention strategies guided by the distinct genomic profile of each RB tumor.

## Methods

This research was conducted under Institutional Review Board approval and adhered to the tenets of the Declaration of Helsinki. Written informed consent was obtained from the participant’s parents prior to inclusion in the study.

### Patient demographics and samples

This report includes one patient with bilateral RB. Relevant clinical information was obtained from retrospective chart reviews. AH samples were extracted via clear corneal paracentesis^15^ during routine intravitreal melphalan (IVM) therapy^22^ or immediately following secondary enucleation.

### Genomic analysis of AH samples

Copy number analysis of AH ctDNA was performed as previously described^10,15,23^. The presence of >1 Mbp SCNAs was noted at 20% deflection from a baseline human genome^15,23^. Constructed whole genome libraries were also used to identify *RB1* pathogenic mutations using a custom hybridization and next-generation sequencing panel^11^. Our hybridization capture panel covered the full length (including introns and exons) of the *RB1* gene, the *MYCN* gene, and all exons of the *BCOR* and *CREBBP* genes. Detected *RB1* single-nucleotide variants (SNVs) were compared to routine clinical peripheral blood *RB1* testing. When the tumor was available, concordance between AH and tumor DNA profiles was calculated^15,24^.

### Determination of ctDNA Tumor Fraction

CtDNA TFx in sequenced AH samples was determined using ichorCNA software according to previously described protocol^11,20,25^.

### Reporting summary

Further information on research design is available in the Nature Research Reporting Summary linked to this article.

## Supplementary information

Supplementary Information

Reporting Summary

## Data Availability

The data presented in this study are available on Sequence Read Archive with BioProject accession number PRJNA732978.
