# Supplementary material for: Inter-eye genomic heterogeneity in bilateral retinoblastoma via aqueous humor liquid biopsy
Source: NPJ Precis Oncol. 2021 Jul 27;5:73. doi: 10.1038/s41698-021-00212-0 (PMC8316348; doi:10.1038/s41698-021-00212-0)
Supplement: Supplementary file 1 — Supplementary Information [file 41698_2021_212_MOESM1_ESM.pdf]

Supplementary Table 1. Patient demographics, clinical outcomes, *RB1* mutational analysis, and somatic copy number alterations (SCNAs)

Case	Eye	Sex	Age at dx (mos)	IIRC Group Class	AJCC	Seeding type at diagnosis	Blood *RB1* mutation	Somatic *RB1* mutation	Total # of AH sam-ples	RB SCNAs					# SCNA (total) per eye	Req'd ENUC?	Reason for ENUC	Time to ENUC after dx (days)	Follow-up (mos)
										1q	2p	6p	13q	16q					
1	OD	F	13	C	cT2b	dust	13q & 16p deletion; 13q14.2-q31.1 and 949kb loss of 16p12.2	13q deletion c.1981C>T (p.R661W), missense mutation	3	↑(1.3)			↓(0.5)	↓(0.7)	3	no	N/A	N/A	32
1	OS	F	13	D	cT2b	sphere	13q & 16p deletion; 13q14.2-q31.1 and 949kb loss of 16p12.2	13q deletion c.1215+1G>A, splice donor variant	2			↑(1.8)	↓(0.5)		2	yes	secondary (recur)	300	32

NOTE: Gains or losses are indicated as ↑ (gain) or ↓ (loss), with amplitude of the change (as ratio to median).
Abbreviations: AJCC, American Joint Committee on Cancer; AH, aqueous humor; dx, diagnosis; ENUC, enucleation; IIRC, International Intraocular Retinoblastoma Classification; mos, months; *RB1*, retinoblastoma tumor suppressor gene; SCNA, somatic copy number alteration.

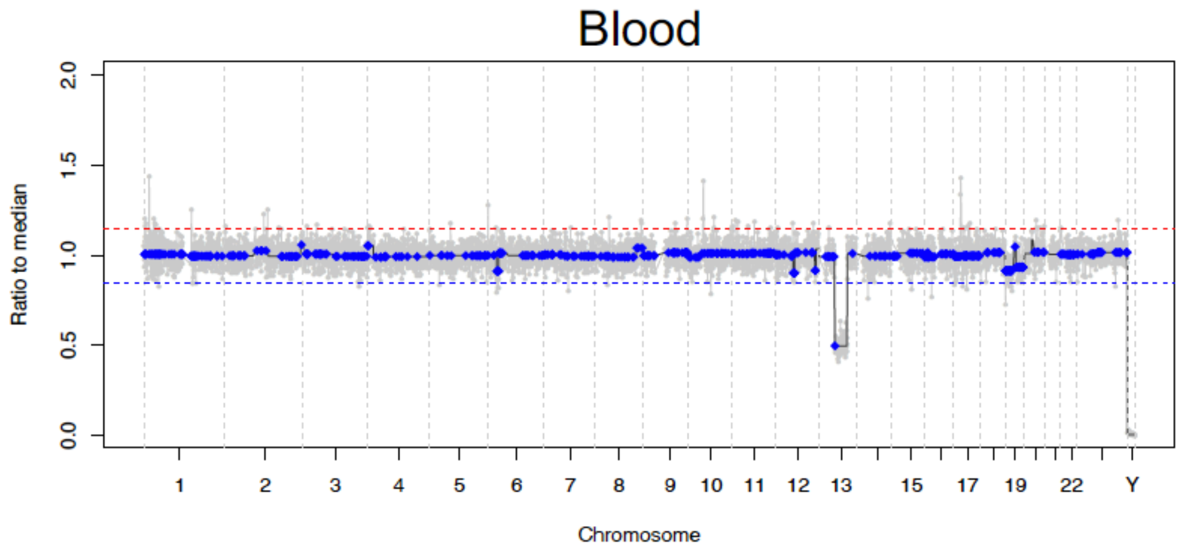

Supplementary Figure 1. The germline 13q deletion is seen on somatic copy number alteration (SCNA) profiling of peripheral blood. Due to germline status, this 13q deletion is seen consistently in all other SCNA profiles. Notably, the germline focal 16p deletion seen on peripheral blood *RB1* testing is not detected in SCNA profiling, as it is below our 1Mbp detection threshold.
