# Supplementary material for: Inter-eye genomic heterogeneity in bilateral retinoblastoma via aqueous humor liquid biopsy
Source: NPJ Precis Oncol. 2021 Jul 27;5:73. doi: 10.1038/s41698-021-00212-0 (PMC8316348; doi:10.1038/s41698-021-00212-0)
Supplement: Supplementary file 2 — Reporting Summary [file 41698_2021_212_MOESM2_ESM.pdf]

nature research

Corresponding author(s): Jesse L. Berry, MD

Last updated by author(s): Mar 15, 2021

Reporting Summary

Nature Research wishes to improve the reproducibility of the work that we publish. This form provides structure for consistency and transparency in reporting. For further information on Nature Research policies, see our Editorial Policies and the Editorial Policy Checklist.

Statistics

For all statistical analyses, confirm that the following items are present in the figure legend, table legend, main text, or Methods section.

n/a	Confirmed	
☒	☐	The exact sample size (*n*) for each experimental group/condition, given as a discrete number and unit of measurement
☒	☐	A statement on whether measurements were taken from distinct samples or whether the same sample was measured repeatedly
☒	☐	The statistical test(s) used AND whether they are one- or two-sided<br>*Only common tests should be described solely by name; describe more complex techniques in the Methods section.*
☒	☐	A description of all covariates tested
☒	☐	A description of any assumptions or corrections, such as tests of normality and adjustment for multiple comparisons
☒	☐	A full description of the statistical parameters including central tendency (e.g. means) or other basic estimates (e.g. regression coefficient) AND variation (e.g. standard deviation) or associated estimates of uncertainty (e.g. confidence intervals)
☒	☐	For null hypothesis testing, the test statistic (e.g. *F*, *t*, *r*) with confidence intervals, effect sizes, degrees of freedom and *P* value noted<br>*Give P values as exact values whenever suitable.*
☒	☐	For Bayesian analysis, information on the choice of priors and Markov chain Monte Carlo settings
☒	☐	For hierarchical and complex designs, identification of the appropriate level for tests and full reporting of outcomes
☒	☐	Estimates of effect sizes (e.g. Cohen's *d*, Pearson's *r*), indicating how they were calculated

Our web collection on statistics for biologists contains articles on many of the points above.

Software and code

Policy information about availability of computer code

Data collection	No software was used for data collection.
Data analysis	ichorCNA software (available at https://github.com/broadinstitute/ichorCNA) is a standard tool for determining cell-free DNA tumor fraction in blood-based liquid biopsies.

For manuscripts utilizing custom algorithms or software that are central to the research but not yet described in published literature, software must be made available to editors and reviewers. We strongly encourage code deposition in a community repository (e.g. GitHub). See the Nature Research guidelines for submitting code & software for further information.

Data

Policy information about availability of data

All manuscripts must include a data availability statement. This statement should provide the following information, where applicable:

- Accession codes, unique identifiers, or web links for publicly available datasets
- A list of figures that have associated raw data
- A description of any restrictions on data availability

Most data generated or analyzed during this study are included in this published article (and its supplementary information files). Additional raw data generated during the current study are available from the corresponding author upon reasonable request.

Field-specific reporting

Please select the one below that is the best fit for your research. If you are not sure, read the appropriate sections before making your selection.

☒ Life sciences ☐ Behavioural & social sciences ☐ Ecological, evolutionary & environmental sciences

For a reference copy of the document with all sections, see nature.com/documents/nr-reporting-summary-flat.pdf

Life sciences study design

All studies must disclose on these points even when the disclosure is negative.

Sample size	5 aqueous humor samples (3 samples from the right eye and 2 samples from the left eye) from one patient with bilateral retinoblastoma.
Data exclusions	No data was excluded.
Replication	Our methods of specimen collection, handling, storage, and processing have been standardized (Berry 2017, Berry 2018, Berry 2019, Xu 2020, Polski 2020). In previously published evaluation of >100 samples, less than 5% of aqueous samples were removed for quality control due to poor reads alignment ratio (Xu, 2020). We have previously reported concentrations of ctDNA isolated from AH samples (median 0.2 ng/µL, range 0.084-56 ng/µL) (Berry, 2018). We have confirmed analytical validity as evidenced by our high concordance rate with the matched tumor tissue from the left eye. In previous work, we have consistently reported concordances >95% (Berry 2017, Berry 2018, Xu 2020) and our methods of ctDNA extraction and sequencing have been standardized (Berry 2017, Berry 2018, Berry 2019, Xu 2020, Polski 2020).
Randomization	This was a case report with a sample size of one patient. Randomization was not applicable to this study.
Blinding	This was a case report with a sample size of one patient. Blinding was not applicable to this study.

Reporting for specific materials, systems and methods

We require information from authors about some types of materials, experimental systems and methods used in many studies. Here, indicate whether each material, system or method listed is relevant to your study. If you are not sure if a list item applies to your research, read the appropriate section before selecting a response.

Materials & experimental systems

n/a	Involved in the study	
☒	☐	Antibodies
☒	☐	Eukaryotic cell lines
☒	☐	Palaeontology and archaeology
☒	☐	Animals and other organisms
☐	☒	Human research participants
☐	☒	Clinical data
☒	☐	Dual use research of concern

Methods

n/a	Involved in the study	
☒	☐	ChIP-seq
☒	☐	Flow cytometry
☒	☐	MRI-based neuroimaging

Human research participants

Policy information about studies involving human research participants

Population characteristics	One 13 month-old female patient with bilateral retinoblastoma with germline 13q and 16p12.2 deletions. The right eye was classified as International Intraocular Retinoblastoma Classification (IIRC) Group C / Stage cT2b; the left eye was Group D / Stage cT2b.
Recruitment	One patient diagnosed with bilateral retinoblastoma in January 2017 at the Children's Hospital Los Angeles (CHLA). Written informed consent was obtained from the participant's parents prior to inclusion in the study.
Ethics oversight	This research was conducted under Institutional Review Board approval and adhered to the tenets of the Declaration of Helsinki.

Note that full information on the approval of the study protocol must also be provided in the manuscript.

Clinical data

Policy information about clinical studies

All manuscripts should comply with the ICMJE guidelines for publication of clinical research and a completed CONSORT checklist must be included with all submissions.

Clinical trial registration	This study was not a clinical trial
Study protocol	This study was not a clinical trial
Data collection	This study was not a clinical trial
Outcomes	This study was not a clinical trial
